# A multiscale dataset for understanding complex eco-hydrological processes in a heterogeneous oasis system

**DOI:** 10.1038/sdata.2017.83

**Published:** 2017-06-27

**Authors:** Xin Li, Shaomin Liu, Qin Xiao, Mingguo Ma, Rui Jin, Tao Che, Weizhen Wang, Xiaoli Hu, Ziwei Xu, Jianguang Wen, Liangxu Wang

**Affiliations:** 1 Cold and Arid Regions Environmental and Engineering Research Institute, Chinese Academy of Sciences, Lanzhou 730000, China; 2 CAS Centre for Excellence in Tibetan Plateau Earth Sciences, Chinese Academy of Sciences, Beijing 100101, China; 3 State Key Laboratory of Earth Surface Processes and Resource Ecology, School of Natural Resources, Faculty of Geographical Science, Beijing Normal University, Beijing 100875, China; 4 State Key Laboratory of Remote Sensing Science, Institute of Remote Sensing Applications, Chinese Academy of Sciences, Beijing 100101, China; 5 School of Geographical Sciences, Southwest University, Beibei, Chongqing 400715, China; 6 Institute of Urban Studies, Shanghai Normal University, Shanghai 200234, China

**Keywords:** Hydrology, Atmospheric science

## Abstract

We introduce a multiscale dataset obtained from Heihe Watershed Allied Telemetry Experimental Research (HiWATER) in an oasis-desert area in 2012. Upscaling of eco-hydrological processes on a heterogeneous surface is a grand challenge. Progress in this field is hindered by the poor availability of multiscale observations. HiWATER is an experiment designed to address this challenge through instrumentation on hierarchically nested scales to obtain multiscale and multidisciplinary data. The HiWATER observation system consists of a flux observation matrix of eddy covariance towers, large aperture scintillometers, and automatic meteorological stations; an eco-hydrological sensor network of soil moisture and leaf area index; hyper-resolution airborne remote sensing using LiDAR, imaging spectrometer, multi-angle thermal imager, and L-band microwave radiometer; and synchronical ground measurements of vegetation dynamics, and photosynthesis processes. All observational data were carefully quality controlled throughout sensor calibration, data collection, data processing, and datasets generation. The data are freely available at figshare and the Cold and Arid Regions Science Data Centre. The data should be useful for elucidating multiscale eco-hydrological processes and developing upscaling methods.

## Background & Summary

The modelling and observation of land-surface system processes must address the scaling issue, which is a complex problem that is intertwined with the nonlinearity and the heterogeneity. Scaling is challenging within all branches of land-surface science, including hydrology^1,2^, ecology^3^, soil science^4^, and boundary layer meteorology^5^, and becomes increasingly prominent with advancements in these fields. It is an urgent need to obtain multiscale observation to further improve our understanding of the scaling issue and validate scaling transformation methods.

However, multi-scale and multidisciplinary data in land-surface science were scarce until the mid-2000s^6^. Since then, data availability has improved and benefited from state-of-the-art *in situ* and remote sensing observations and data acquisition techniques. Moreover, multi-scale land surface observation experiments have been implemented globally within the last decade^7–9^. These experiments have provided a promising method for bridging knowledge gaps among microscopic-, mesoscopic- and macroscopic-scale understanding.

The Heihe Watershed Allied Telemetry Experimental Research (HiWATER) project is an example of such experiments^10^. HiWATER is a simultaneous airborne, satellite-borne and ground-based eco-hydrological experiment designed from an interdisciplinary perspective. This project was initialized within the framework of the Integrated Research of the Eco-hydrological Processes of the Heihe River Basin (HRB), which is a major research program supported by the National Natural Science Foundation of China^11,12^. This program addresses scaling issues associated with eco-hydrological processes through process study, modelling, and observation. HiWATER focuses on obtaining multi-scale observation data to support the scaling studies of this major research program. In HiWATER, scaling is considered a grand challenge in two aspects. (1) Upscaling in situ observations to a scale of approximately 1 kilometre, which is consistent with medium spatial resolution remote sensing as well as river basin-scale eco-hydrological models. Then, the upscaled ground truth can be used to validate remote sensing products and model results on heterogeneous land surfaces and quantify the uncertainty associated with heterogeneity^13^. (2) Use the multiscale data in understanding key eco-hydrological processes across multiple scales, including leaf, individual plant, community, landscape, watershed, and river basin. Therefore, preconditions include obtaining multi-scale observations with sufficiently high spatial and temporal resolution and providing data for disciplines, such as hydrology, ecology, and boundary layer meteorology.

HiWATER was implemented in the HRB, the second largest inland river basin in China, which has diverse landscapes, environmental extremes (mountain cryosphere and arid environments), and conflicting interests (economic development and ecosystem restoration). Additionally, the HRB is an experimental river basin that has been used for hydrological, ecological, and integrated studies for over 30 years^11^. HiWATER lasts from 2012–2016. Several intensive observation periods (IOPs) and continuous hydrometeorological observations were carried out during HiWATER. Only data collected during the IOP in 2012 are presented in this paper. The 2012 IOP was implemented during the growing season from April to September in an oasis with surrounding deserts located in the midstream area of the HRB^10^.

HiWATER 2012 IOP datasets were released after careful quality control throughout sensor calibration, observation, data collection, data processing, and dataset generation. The datasets have been made available to the scientific community through figshare. Additionally, the datasets can also be downloaded from the Cold and Arid Regions Science Data Centre at Lanzhou (CARD), a member of the World Data System. Metadata are available in both English and Chinese, with the digital object identifier (doi) and data citation attached to each dataset.

## Methods

### Experimental design and data acquisition

The 2012 IOP of HiWATER occurred in an oasis and surrounding deserts located in the midstream area of the HRB. Complex energy and water exchanges between oases and surrounding deserts exist on the river basin scale, which differ sharply in landscape as well as in hydrological and thermal conditions^14,15,16^. The widely distributed farmland shelterbelts and irrigation scheduling within the oasis can result in small-scale kinetic and thermal heterogeneities, respectively. Obviously, only observing land-surface variables at limited sites cannot capture the heterogeneities of the abovementioned processes. Hence, full coverage of the following spatial scales must be achieved to understand the complex eco-hydrological processes within the system:

- River basin scale (oasis-desert system): Tens of thousands of square kilometres.
- Irrigation district scale: A sub-unit of the river basin, usually tens to hundreds of square kilometres.
- Kilometre scale: Corresponds to medium spatial resolution remote sensing (such as Moderate Resolution Imaging Spectroradiometer (MODIS), MEdium Resolution Imaging Spectrometer (MERIS), and Chinese meteorological satellite FY-3) and river basin-scale hydrological and ecological models. The footprint of a large-aperture scintillometer (LAS) matches this scale.
- Landscape scale: Corresponds to farmland plots surrounded by shelterbelts, usually a few to tens of hectares. The footprint of an eddy covariance (EC) system corresponds to this scale.
- Metre to sub-metre scale: The footprints of most soil moisture and leaf area index (LAI) sensors correspond to this scale.
- Individual plant scale.
- Leaf scale.
- Stomatal scale: The scale of plant stomata.

**Figure 1:**
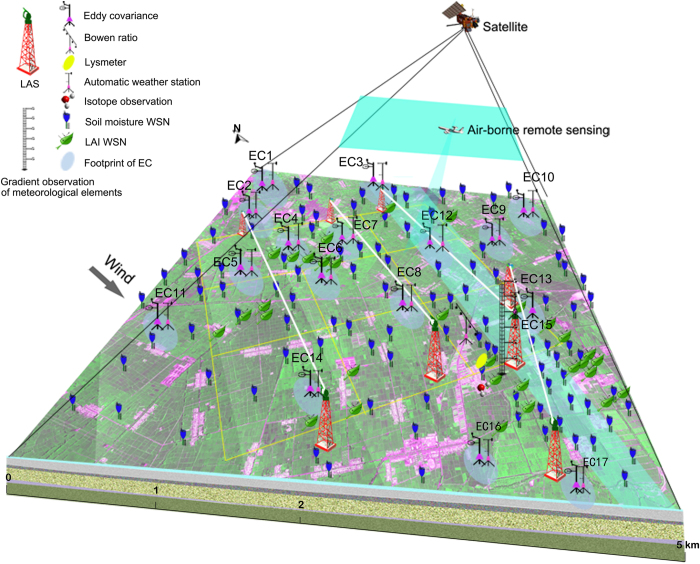
Instrumentation in the HiWATER intensive observation period in 2012 to capture the land surface heterogeneity.

Instruments for the oasis-desert system were arranged in hierarchically nested scales to capture multi-scale eco-hydrological processes. We established a sparse network to investigate the oasis-desert interaction. One superstation was constructed within the oasis, and four EC towers and four two-layer automatic meteorological stations (AMSs) were installed in different landscapes surrounding the oasis, including sandy desert, desert pavement, desert steppe, and wetland. All components of surface energy and water balances and associated near-surface atmospheric states were measured to capture the heterogeneity of the water and energy cycle in the oasis-desert system (Fig. 1). Additionally, several airborne remote sensing missions covered this area.

Intensive observations were implemented at the irrigation district scale. This foci experimental area (FEA) spans approximately 5.5×5.5 km^2^, which is a fragmented landscape occupied primarily by seed corn. Other crops or land use types include vegetable, orchard, and shelterbelt. The precipitation in this area is low, with approximately 150 mm yr^−1^, and irrigation water is withdrawn from streamflow and groundwater. The FEA was equipped with a flux observation matrix of 17 EC towers and AMSs; 4 LAS pairs^17,18^; and an eco-hydrological sensor network of soil moisture with 180 sensor nodes^19^ and leaf area index (LAI) with 42 sensor nodes^20^. Other *in situ* observations include stable isotope measurements of evapotranspiration (ET)^21^, Cosmic-Ray probe soil moisture (COSMOS) measurements, sap flow, irrigation water, photosynthesis, soil respiration, stomatal conductance, vegetation dynamics (LAI, fraction of photosynthetically active radiation (fpar), vegetation coverage, vegetation/crop type, vegetation height, and phenology), emissivity, reflectance, atmospheric profiles of humidity and temperature, and aerosol optical depth^22^. Additionally, soil samples were collected and soil properties such as texture and thermal and hydraulic parameters were analysed in a laboratory. A total of 12 airborne remote sensing missions were conducted to cover the FEA using LiDAR, an imaging spectrometer, a multi-angle thermal imager, and an L-band microwave radiometer. Calibration and validation of airborne remote sensing were completed using the abovementioned ground observations and supplemented through intensive tasks designed to measure target variables on the ground. The instrumentation listed above is illustrated in Fig. 1. The sensors used in HiWATER are summarized in Table 1 (available online only), and the airborne remote sensors are detailed in Table 2. Satellite remote sensing data at different resolutions and from various satellite sensors were acquired through data sharing programmes and commercial purchases. The satellite data were archived with other HiWATER datasets.

Spatial scale is explicitly considered for all observations. The matrix of EC towers, AMS, and LAS was designed to fully encompass all landscapes in the oasis and to form true multi-scale observations. Observational footprints overlapped with landscape to kilometre scales, and the measurement foci included ET, sensible heat flux, radiation fluxes, and soil heat flux to close the energy balance. The soil moisture and LAI sensor network was designed to form an unbiased estimation from sub-metre to kilometre scales using geostatistical model-based sampling methods^23^. The design principles of the sampling included best linear unbiased estimation, multi-scale variation acquisition, cost effectiveness, and implementation feasibility. Ground-based observations of vegetation dynamics, photosynthesis, and soil respiration were completed at individual plant, leaf, and stomatal scales. These observations were designed with considerations of sampling different crops and scaling up to a resolution of approximately 1 kilometre so that the scaled values could be compared with satellite remote sensing products and used in river basin eco-hydrological modelling. Airborne remote sensing was deigned to bridge the scale gap between *in situ* and satellite remote sensing^24^. All airborne sensors' resolutions were at least one order of magnitude higher than those of satellite remote sensing. Therefore, high-resolution products, such as digital elevation model, land cover map, albedo, LAI, crop height, land surface temperature (LST), and soil moisture, could be derived to reveal landscape and thermal heterogeneities.

The use of multi-scale observations following multiple approaches and using different instruments is a concern in experiment design. On the ground, this strategy focused on ET and soil moisture. Stable isotope and sap flow methods were used to fill the gap in measuring ET processes at stomatal, leaf, individual plant, and metre scales and to separate evaporation and transpiration. The soil moisture sensor network is a nested and cross-scale observational approach because point measurements will be upscaled to gridded data with resolutions from 30 to 1,000 metres. Furthermore, the soil moisture sensor network was supplemented by AMSs and flux towers with soil moisture profile measurements up to approximately −1.0 to −1.6 metres in depth, COSMOS with a 350-metre radius, ground-penetrating radar, and manual observations of soil moisture at a fine scale. Additionally, optical and microwave sensors used concurrently in some airborne missions and multi-resolution, multi-angular, and multi-source airborne data were obtained by flying at different heights. These data are particularly useful in developing and validating upscaling methods.

The temporal density of automatic systems, such as sensor networks and AMS, was up to one minute. Typically, sampling frequencies were 10 to 30 min so that the temporal resolution was sufficiently high to capture temporal dynamics and analyse temporal stationarity.

### Data quality control

**Figure 2:**
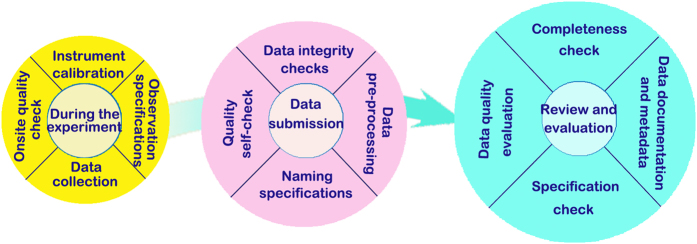
Data quality control in HiWATER data collection, processing, archiving and sharing.

Data quality control is a last-for-ever process in HiWATER. Before, during, and after the field observation, data processing, dataset generation, and data release, a series of quality control measures were undertaken, which were implemented through the following procedures (Fig. 2).

(1) Experiment preparation period. Observation instrument operating specifications were formulated, and observers were trained. In addition, instrument selection, alignment and calibration were completed to ensure appropriate implementation of operating specifications as well as the accuracy and consistency of the observation instruments.

(2) Experimental IOPs. The integrity of observation information and data quality were achieved through the implementation of operation specifications, technical inspections, instrument alignment and calibration, and maintenance of detailed experimental procedure records and an experimental logbook, among other measures.

(3) Data collection. Data accuracy and integrity were achieved through data integrity checks, quality self-examination, and standardized data file naming.

(4) Data processing. Standard data processing procedures were performed. A thematic data processing group was established and was dedicated to data processing. Organized discussions and studies were employed to assess difficulties and problems associated with the processing of key datasets.

(5) Writing and reviews of the metadata. Many measures were adopted during this period, including metadata and raw observation data consistency checks, supplementation of standardized descriptive data information, data integrity and accessibility checks, missing data supplementation during collection and digitization, and invalid data investigation. After the collation of the metadata, numerous peer-review cycles were implemented to improve the metadata quality.

(6) Expert review. Peer-review methods were used to ensure HiWATER dataset quality. First, experts of thematic experimental observations, such as flux observation matrix, sensor network observations, and airborne remote sensing, completed internal quality reviews and crosschecks. Second, experts in the relevant fields performed data quality analyses, including data checks, data availability suggestions and overall data quality evaluations.

(7) Data users appraisal stage. Data issues were promptly corrected according to the advice given by data users after dataset release in order to improve data quality and services.

## Data Records

HiWATER 2012 IOP data were organized according to the flux observation matrix, the eco-hydrological sensor network, other ground-based observations, and airborne missions and airborne remote sensing products. A total of 102 datasets were generated formally. Dataset quantities, sizes, and formats are summarized in Table 3. Detailed information, including title, observation variable, location, observation time, sensor or instrument used, quality control, spatial and temporal scales, and the doi of each dataset, is provided in Table 1 (available online only). High-resolution satellite remote sensing data from VNIR, thermal infrared (TIR), synthetic aperture radar (SAR), and LiDAR sensors were obtained via data sharing programmes and limited commercial purchases. Additionally, we archived the satellite remote sensing data in the HiWATER data repository (Table 4). However, the copyrights of these satellite remote sensing data belong to the original data providers, so we cannot release them as HiWATER datasets but can offer them to users in an offline mode.

The data obtained from the flux observation network, ecohydrological sensor network, and other ground-based observations are publicly and freely downloadable from figshare (Data Citation 1) and the HiWATER data repository in the CARD ( Data Citation 2–85), in which more detailed information including data citation, related publications, background introduction, and relationship with other datasets is available. As for the airborne remote sensing data, the L-band microwave radiometer data and the soil moisture data products derived from these data are also available online for users’ direct download (Data Citation 103). In total, 85 HiWATER IOP datasets are fully and freely downloadable at figshare as a whole dataset and at the CARD as individual datasets. However, in accordance with the laws and regulations in China, the hyper-resolution remote sensing data, including those from LiDAR, imaging spectrometer, and multi-angle thermal imager, cannot be placed online. Therefore, these datasets (Data Citation 86–86–Data Citation 87–87–Data Citation 88–88–Data Citation 89–89–Data Citation 90–90–Data Citation 91–91–Data Citation 92–92–Data Citation 93–93–Data Citation 94–94–Data Citation 95–95–Data Citation 96–96–Data Citation 97–97–Data Citation 98–98–Data Citation 99–99–Data Citation 100–100–Data Citation 101–101–102) are offered in an offline mode at the CARD. The users can submit a data application form on line via the HiWATER data system. Once the application is approved, the data will be sent to the user.

**Figure 3:**
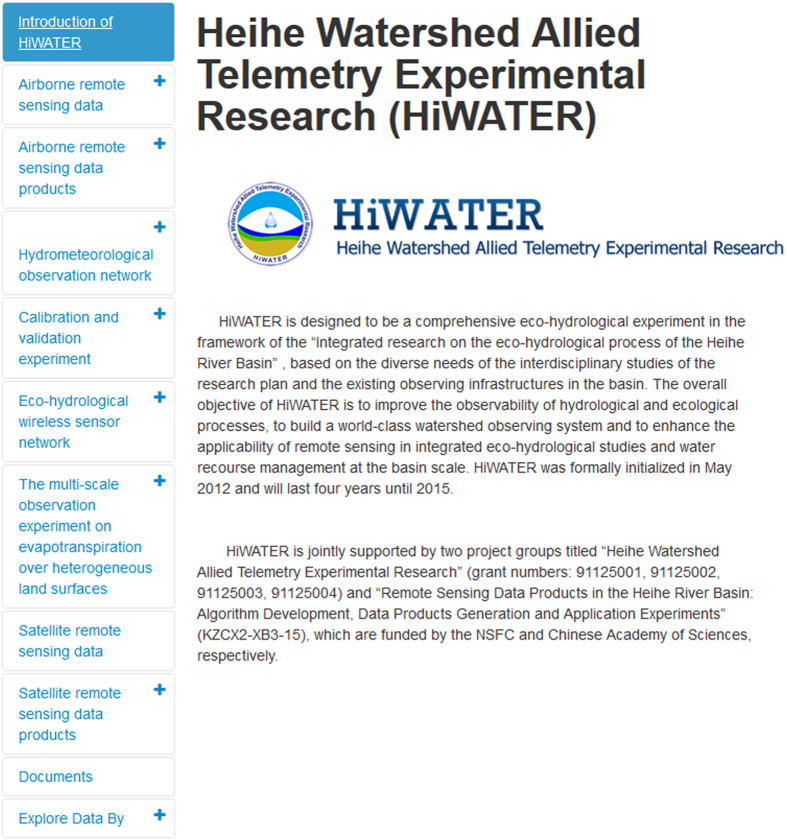
The navigation web page of HiWATER data repository at the Cold and Arid Regions Science Data Centre.

Additionally, special navigation web pages were built on CARD to browse, navigate, search, and download HiWATER data (http://card.westgis.ac.cn/hiwater) (Fig. 3). Users can find the datasets via a keyword search, classified navigation or theme-based exploration (e.g., by timeline, map, author, or thumbnail), which are offered by the metadata database. The ISO 19,115 geographic metadata standard was used to describe the HiWATER data. All metadata are available in both English and Chinese. Additionally, doi and data citation information are attached to each data record. The unique doi of a dataset will lead the user to a web page that provides a detailed data description and a data download URL for the individual dataset. The data are redistributed by a File Transfer Protocol (FTP) server with an auto-generated FTP account.

## Technical Validation

Sensor calibration, measurement validation and other quality control measures are prerequisites to ensuring data quality in HiWATER. We describe the quality control measures in the Methods section. Sensor and instrument calibration was conducted as follows.

The calibration of EC, AMS, and LAS systems is summarized in data records 1–50 in Table 1 (available online only). The 20 EC system sets, 7 LAS sets, and 18 radiometer sets used in the experiment were compared under a flat Gobi desert surface prior to the 2012 HiWATER IOP during May 14–24, 2012. The results indicate that all ECs, LASs and radiometers were consistent. Compared to the reference instrument, the average root-mean-square error (RMSE) and mean relative error (MRE) of the sensible heat flux measured by the EC were 13.00 W m^−2^ and −2.02% and 4.47 W m^−2^ and 0.11% for latent heat flux, respectively. The average RMSE and MRE values for the LAS were 10.26 W m^−2^ and 5.48%, respectively. The RMSE and MRE for net radiation were 10.38 W m^−2^ and 1.24%, respectively. The EC and LAS measurements were consistent with a regression slope of less than 8%, which indicated reliability during HiWATER. The comparison results were consistent or better than the previous comparison results in the international experiments^18^. Additionally, the sensors of wind speed, air temperature and humidity profiles at the superstation and soil temperature and moisture profile at each site were intercompared as well prior the 2012 IOP.

The calibrations of soil moisture, LST, and LAI sensors used in the sensor network are described in data records 51–54 in Table 1 (available online only). The sensor network employed a large number of different sensors. Soil moisture sensors included 200 SPADE and 150 Hydra Probe II. For reliability and efficiency, the accuracy and consistency of each sensor were calibrated using the two-point calibration method with desert sand and saturated soil samples as dry and wet points, respectively. Then, the oven-drying method was used to evaluate measurement accuracy. The calibration results indicated that the consistency between the same type of sensors is greater than 95%. The accuracies of soil moisture for SPADE and Hydra Probe II are 0.032 and 0.011 m^3^ m^−3^, respectively. The LST sensor, SI-111, was calibrated using the BDB blackbody calibrator at a constant temperature of 23 °C and a water-ice mixture at 0 °C. The accuracy of the LST measured by the SI-111 sensor was within 0.15 °C^19^. The LAI instrument used in the WSN, LAINet, was compared with LAI-2000, a commercial instrument used to measure LAI. Consistency was relatively high at LAI<3.5. However, LAINet could capture the dynamics at LAI >3.5, whereas the LAI-2000 measurements were saturated, indicating an improved accuracy of the LAINet measurements over those of LAI-2000^20^.

Many instruments were used in other ground measurements. Most instruments were calibrated using absolute and cross-calibration strategies (data records 55–76). The field spectrometers, including ASD and Spectra Vista Corp, were cross-calibrated with each other. The black board and old white board were calibrated according to a new standard white board. The GPS radiosondes, including Changfeng and Vaisala, were also cross-calibrated using a conventional radiosonde. The results indicated close agreement among different radiosonde measurements. Two CE-318 sun photometers used in HiWATER were cross-calibrated in June 15–16, 2012. The Scintec Flat Array Sodar, which was used to measure wind direction, wind speed, and disturbance characteristics in the lower atmosphere, was cross-calibrated with the wind profile data obtained at the Daman Superstation, a 40-metre boundary layer tower. The results indicated close agreement between these two types of wind profile measurements. Self-recording point thermometers and handheld infrared thermometers were used to measure LST in HiWATER. All sensors were absolutely calibrated at constant temperatures from 0 to 60 °C with a 5 °C interval. Calibration was repeated five times for each temperature. The calibration experiments indicate that the temperature accuracies of a majority of the sensors are less than 1 K.

Airborne remote sensing instrument calibration and data validation are summarized in data records 77–102 in Table 1 (available online only). The radiometric parameter of the VIS/NIR sensor was calibrated in the calibration laboratory of the Institute of Remote Sensing and Digital Earth, Chinese Academy of Sciences, using an integrating sphere as the light source, which was developed by the Labsphere Corporation. Additionally, the wavelength was calibrated using a monochromator. An EO-1 blackbody was used to calibrate the radiometric uniformity and temperature of the thermal infrared sensor. The geometric correction of the frame sensor was performed using a specifically designed three-dimensional target. A bundle-adjustment procedure was completed for LiDAR calibration to characterize linear spatial displacements between the IMU and the sensing array. The L-band microwave radiometer, PLMR was calibrated using the two-end calibration method prior to and following mounting on the aircraft in each flight. The warm-end calibration adopted a closed blackbody box with the environmental temperature measured using 16 thermal sensors, whereas the cold end was calibrated by measuring the sky brightness temperature (Tb). The largest reservoir in the study area was selected as a calibration reference. The temperature of the top water layer was measured every minute in the experimental period. The Tbs over the reservoir were measured during each flight mission. Therefore, the measured Tb over a water body can be compared to the Tbs calculated by the radiative transfer model of water. The two-end calibration and water body reference indicated that the accuracy of measurements at a small incidence angle was superior to that at a large incidence angle, and the average accuracy was superior to 1.0 K for both of the vertical and horizontal polarizations. Caution should be taken when using Tb data because the radio frequency interference contamination was sometime higher than expected at v-polarization.

Airborne and satellite remote sensing products were quantitatively validated using simultaneous *in situ* observations, with a particular focus on upscaling point- and footprint-scale observations to the pixel scale^22,25^. The overall quality of remote sensing data products was evaluated based on the accuracy and uncertainty, and this information was made available in the metadata of the data products. A high-quality remote sensing data product was released only when its accuracy was higher than the required standard threshold. Otherwise, the algorithm was improved and then re-executed for product generation until a satisfactory accuracy was reached.

## Additional Information

**How to cite this article:** Li, X. *et al.* A multiscale dataset for understanding complex eco-hydrological processes in a heterogeneous oasis system. *Sci. Data* 4:170083 doi: 10.1038/sdata.2017.83 (2017).

**Publisher’s note:** Springer Nature remains neutral with regard to jurisdictional claims in published maps and institutional affiliations.

## Supplementary Material



## Figures and Tables

**Table 1:** Summarizations of data, measurement instruments, quality control, and data availability

**ID**	**Dataset**	**Subject**	**Sub-subject**	**Observation variable**	**Place/location**	**Observation time**	**Sensor/Instrument**	**Quality control**	**Spatial scale/resolution**	**Temporal scale/resoluton**	**DOI**
1	HiWATER: The Multi-Scale Observation Experiment on Evapotranspiration over heterogeneous land surfaces (MUSOEXE) Dataset—flux observation matrix (an automatic weather station of Daman Superstation)	Matrix	Meteorological gradient observation systems	Note 1	Daman superstation (No. 15 station in matrix)	2012-05-10 to 2012-09-26	Note 2	Intercomparison of the same type sensors in the same level/height	Landscape scale	10 min (note 4)	10.3972/hiwater.073.2013.db
2	HiWATER: The Multi-Scale Observation Experiment on Evapotranspiration over heterogeneous land surfaces (MUSOEXE) Dataset—flux observation matrix (an automatic weather station at Bajitan Gobi station)		AMS	Note 3	Bajitan gobi desert station	2012-05-13 to 2012-09-21		Intercomparison of radiometer, soil temperature and moisture sensors, with other sensors carefully checked	Landscape scale	10 or 30 min	10.3972/hiwater.076.2013.db
3	HiWATER: The Multi-Scale Observation Experiment on Evapotranspiration over heterogeneous land surfaces (MUSOEXE) Dataset—flux observation matrix (an automatic weather station at Huazhaizi desert steppe station)			Note 3	Huazhaizi desert steppe station	2012-06-02 to 2012-09-21		Intercomparison of radiometer, soil temperature and moisture sensors, with other sensors carefully checked	Landscape scale	10 min	10.3972/hiwater.078.2013.db
4	HiWATER: The Multi-Scale Observation Experiment on Evapotranspiration over heterogeneous land surfaces (MUSOEXE) Dataset—flux observation matrix (an automatic weather station at Shenshawo sandy desert station)			Note 3	Shenshawo sandy desert station	2012-06-01 to 2012-09-21		Intercomparison of radiometer, soil temperature and moisture sensors, with other sensors carefully checked	Landscape scale	10 min	10.3972/hiwater.077.2013.db
5	HiWATER: The Multi-Scale Observation Experiment on Evapotranspiration over heterogeneous land surfaces (MUSOEXE) Dataset—flux observation matrix (an automatic weather station at Zhangye wetland station)			Note 3	Zhangye wetland station	2012-06-25 to 2012-09-21		Intercomparison of radiometer, soil temperature and moisture sensors, with other sensors carefully checked	Landscape scale	10 min	10.3972/hiwater.079.2013.db
6	HiWATER: The Multi-Scale Observation Experiment on Evapotranspiration over heterogeneous land surfaces (MUSOEXE) Dataset—flux observation matrix (an automatic weather station of site No.1)			Note 3	No. 1 station in matrix	2012-06-10 to 2012-09-17		Intercomparison of radiometer, soil temperature and moisture sensors, with other sensors carefully checked	Landscape scale	10 min	10.3972/hiwater.059.2013.db
7	HiWATER: The Multi-Scale Observation Experiment on Evapotranspiration over heterogeneous land surfaces (MUSOEXE) Dataset—flux observation matrix (an automatic weather station of site No.2)			Note 3	No. 2 station in matrix	2012-05-03 to 2012-09-21		Intercomparison of radiometer, soil temperature and moisture sensors, with other sensors carefully checked	Landscape scale	10 min	10.3972/hiwater.060.2013.db
8	HiWATER: The Multi-Scale Observation Experiment on Evapotranspiration over heterogeneous land surfaces (MUSOEXE) Dataset—flux observation matrix (an automatic weather station of site No.3)			Note 3	No. 3 station in matrix	2012-06-03 to 2012-09-18		Intercomparison of radiometer, soil temperature and moisture sensors, with other sensors carefully checked	Landscape scale	10 min	10.3972/hiwater.061.2013.db
9	HiWATER: The Multi-Scale Observation Experiment on Evapotranspiration over heterogeneous land surfaces (MUSOEXE) Dataset—flux observation matrix (an automatic weather station of site No.4)			Note 3	No. 4 station in matrix	2012-05-10 to 2012-09-17		Intercomparison of radiometer, soil temperature and moisture sensors, with other sensors carefully checked	Landscape scale	10 min	10.3972/hiwater.062.2013.db
10	HiWATER: The Multi-Scale Observation Experiment on Evapotranspiration over heterogeneous land surfaces (MUSOEXE) Dataset—flux observation matrix (an automatic weather station of site No.5)			Note 3	No. 5 station in matrix	2012-06-04 to 2012-09-18		Intercomparison of radiometer, soil temperature and moisture sensors, with other sensors carefully checked	Landscape scale	10 min	10.3972/hiwater.063.2013.db
11	HiWATER: The Multi-Scale Observation Experiment on Evapotranspiration over heterogeneous land surfaces (MUSOEXE) Dataset—flux observation matrix (an automatic weather station of site No.6)			Note 3	No. 6 station in matrix	2012-05-09 to 2012-09-21		Intercomparison of radiometer, soil temperature and moisture sensors, with other sensors carefully checked	Landscape scale	10 min	10.3972/hiwater.064.2013.db
12	HiWATER: The Multi-Scale Observation Experiment on Evapotranspiration over heterogeneous land surfaces (MUSOEXE) Dataset—flux observation matrix (an automatic weather station of site No.7)			Note 3	No. 7 station in matrix	2012-05-28 to 2012-09-18		Intercomparison of radiometer, soil temperature and moisture sensors, with other sensors carefully checked	Landscape scale	10 min	10.3972/hiwater.065.2013.db
13	HiWATER: The Multi-Scale Observation Experiment on Evapotranspiration over heterogeneous land surfaces (MUSOEXE) Dataset—flux observation matrix (an automatic weather station of site No.8)			Note 3	No. 8 station in matrix	2012-05-14 to 2012-09-21		Intercomparison of radiometer, soil temperature and moisture sensors, with other sensors carefully checked	Landscape scale	10 min	10.3972/hiwater.066.2013.db
14	HiWATER: The Multi-Scale Observation Experiment on Evapotranspiration over heterogeneous land surfaces (MUSOEXE) Dataset—flux observation matrix (an automatic weather station of site No.9)			Note 3	No. 9 station in matrix	2012-06-04 to 2012-09-17		Intercomparison of radiometer, soil temperature and moisture sensors, with other sensors carefully checked	Landscape scale	10 min	10.3972/hiwater.067.2013.db
15	HiWATER: The Multi-Scale Observation Experiment on Evapotranspiration over heterogeneous land surfaces (MUSOEXE) Dataset—flux observation matrix (an automatic weather station of site No.10)			Note 3	No. 10 station in matrix	2012-06-01 to 2012-09-17		Intercomparison of radiometer, soil temperature and moisture sensors, with other sensors carefully checked	Landscape scale	10 min	10.3972/hiwater.068.2013.db
16	HiWATER: The Multi-Scale Observation Experiment on Evapotranspiration over heterogeneous land surfaces (MUSOEXE) Dataset—flux observation matrix (an automatic weather station of site No.11)			Note 3	No. 11 station in matrix	2012-06-02 to 2012-09-18		Intercomparison of radiometer, soil temperature and moisture sensors, with other sensors carefully checked	Landscape scale	10 min	10.3972/hiwater.069.2013.db
17	HiWATER: The Multi-Scale Observation Experiment on Evapotranspiration over heterogeneous land surfaces (MUSOEXE) Dataset——flux observation matrix (an automatic weather station of site No.12)			Note 3	No. 12 station in matrix	2012-05-10 to 2012-09-21		Intercomparison of radiometer, soil temperature and moisture sensors, with other sensors carefully checked	Landscape scale	10 min	10.3972/hiwater.070.2013.db
18	HiWATER: The Multi-Scale Observation Experiment on Evapotranspiration over heterogeneous land surfaces (MUSOEXE) Dataset—flux observation matrix (an automatic weather station of site No.13)			Note 3	No. 13 station in matrix	2012-05-06 to 2012-09-20		Intercomparison of radiometer, soil temperature and moisture sensors, with other sensors carefully checked	Landscape scale	10 min	10.3972/hiwater.071.2013.db
19	HiWATER: The Multi-Scale Observation Experiment on Evapotranspiration over heterogeneous land surfaces (MUSOEXE) Dataset—flux observation matrix (an automatic weather station of site No.14)			Note 3	No. 14 station in matrix	2012-05-06 to 2012-09-21		Intercomparison of radiometer, soil temperature and moisture sensors, with other sensors carefully checked	Landscape scale	10 min	10.3972/hiwater.072.2013.db
20	HiWATER: The Multi-Scale Observation Experiment on Evapotranspiration over heterogeneous land surfaces (MUSOEXE) Dataset—flux observation matrix (an automatic weather station of site No.16)			Note 3	No. 16 station in matrix	2012-06-01 to 2012-09-17		Intercomparison of radiometer, soil temperature and moisture sensors, with other sensors carefully checked	Landscape scale	10 min	10.3972/hiwater.074.2013.db
21	HiWATER: The Multi-Scale Observation Experiment on Evapotranspiration over heterogeneous land surfaces (MUSOEXE) Dataset—flux observation matrix (an automatic weather station of site No.17)			Note 3	No. 17 station in matrix	2012-05-12 to 2012-09-17		Intercomparison of radiometer, soil temperature and moisture sensors, with other sensors carefully checked	Landscape scale	10 min	10.3972/hiwater.075.2013.db
22	HiWATER: The Multi-Scale Observation Experiment on Evapotranspiration over heterogeneous land surfaces (MUSOEXE) Dataset- flux observation matrix (an eddy covariance system at the Daman Superstation in the lowest layer)		EC	Sensible heat, latent heat, and CO2 fluxes	Daman superstation (No. 15 station in matrix)	2012-05-25 to 2012-09-15	CSAT3 & Li7500A	Calibration and sensor intercomparison	Landscape scale	30 min	10.3972/hiwater.096.2013.db
23	HiWATER: The Multi-Scale Observation Experiment on Evapotranspiration over heterogeneous land surfaces (MUSOEXE) Dataset- flux observation matrix (an eddy covariance system at the Daman Superstation in the highest layer)			Sensible heat, latent heat, and CO2 fluxes	Daman superstation (No. 15 station in matrix)	2012-05-30 to 2012-09-15	CSAT3 & Li7500A	Calibration and sensor intercomparison	Landscape scale	30 min	10.3972/hiwater.097.2013.db
24	HiWATER: The Multi-Scale Observation Experiment on Evapotranspiration over heterogeneous land surfaces (MUSOEXE) Dataset- flux observation matrix (an eddy covariance system at the Bajitan Gobi station)			Sensible heat, latent heat, and CO2 fluxes	Bajitan gobi desert station	2012-05-31 to 2012-09-15	CSAT3 & Li7500	Calibration and sensor intercomparison	Landscape scale	30 min	10.3972/hiwater.098.2013.db
25	HiWATER: The Multi-Scale Observation Experiment on Evapotranspiration over heterogeneous land surfaces (MUSOEXE) Dataset- flux observation matrix (an eddy covariance system at Huazhaizi desert steppe Station)			Sensible heat, latent heat, and CO2 fluxes	Huazhaizi desert steppe Station	2012-06-06 to 2012-09-15	CSAT3 & Li7500	Calibration and sensor intercomparison	Landscape scale	30 min	10.3972/hiwater.100.2013.db
26	HiWATER: The Multi-Scale Observation Experiment on Evapotranspiration over heterogeneous land surfaces (MUSOEXE) Dataset- flux observation matrix (an eddy covariance system at Shenshawo sandy desert Station)			Sensible heat, latent heat, and CO2 fluxes	Shenshawo sandy desert station	2012-06-01 to 2012-09-15	CSAT3 & Li7500	Calibration and sensor intercomparison	Landscape scale	30 min	10.3972/hiwater.099.2013.db
27	HiWATER: The Multi-Scale Observation Experiment on Evapotranspiration over heterogeneous land surfaces (MUSOEXE) Dataset—flux observation matrix (an eddy covariance system at Zhangye wetland Station)			Sensible heat, latent heat, and CO2 fluxes	Zhangye wetland station	2012-06-25 to 2012-09-26	Gill & Li7500A	Calibration and sensor intercomparison	Landscape scale	30 min	10.3972/hiwater.101.2013.db
28	HiWATER: The Multi-Scale Observation Experiment on Evapotranspiration over heterogeneous land surfaces (MUSOEXE) Dataset—flux observation matrix (an eddy covariance system of site No.1)			Sensible heat, latent heat, and CO2 fluxes	No. 1 station in matrix	2012-06-04 to 2012-09-17	Gill & Li7500A	Calibration and sensor intercomparison	Landscape scale	30 min	10.3972/hiwater.080.2013.db
29	HiWATER: The Multi-Scale Observation Experiment on Evapotranspiration over heterogeneous land surfaces (MUSOEXE) Dataset—flux observation matrix (an eddy covariance system of site No.2)			Sensible heat, latent heat, and CO2 fluxes	No. 2 station in matrix	2012-06-03 to 2012-09-21	CSAT3 & Li7500A	Calibration and sensor intercomparison	Landscape scale	30 min	10.3972/hiwater.081.2013.db
30	HiWATER: The Multi-Scale Observation Experiment on Evapotranspiration over heterogeneous land surfaces (MUSOEXE) Dataset—flux observation matrix (an eddy covariance system of site No.3)			Sensible heat, latent heat, and CO2 fluxes	No. 3 station in matrix	2012-06-03 to 2012-09-18	Gill & Li7500A	Calibration and sensor intercomparison	Landscape scale	30 min	10.3972/hiwater.082.2013.db
31	HiWATER: The Multi-Scale Observation Experiment on Evapotranspiration over heterogeneous land surfaces (MUSOEXE) Dataset—flux observation matrix (an eddy covariance system of site No.4)			Sensible heat, latent heat, and CO2 fluxes	No. 4 station in matrix	2012-05-31 to 2012-09-17	CSAT3 & Li7500A	Calibration and sensor intercomparison	Landscape scale	30 min	10.3972/hiwater.083.2013.db
32	HiWATER: The Multi-Scale Observation Experiment on Evapotranspiration over heterogeneous land surfaces (MUSOEXE) Dataset—flux observation matrix (an eddy covariance system of site No.5)			Sensible heat, latent heat, and CO2 fluxes	No. 5 station in matrix	2012-06-03 to 2012-09-18	CSAT3 & Li7500	Calibration and sensor intercomparison	Landscape scale	30 min	10.3972/hiwater.084.2013.db
33	HiWATER: The Multi-Scale Observation Experiment on Evapotranspiration over heterogeneous land surfaces (MUSOEXE) Dataset—flux observation matrix (an eddy covariance system of site No.6)			Sensible heat, latent heat, and CO2 fluxes	No. 6 station in matrix	2012-05-28 to 2012-09-21	CSAT3 & Li7500A	Calibration and sensor intercomparison	Landscape scale	30 min	10.3972/hiwater.085.2013.db
34	HiWATER: The Multi-Scale Observation Experiment on Evapotranspiration over heterogeneous land surfaces (MUSOEXE) Dataset—flux observation matrix (an eddy covariance system of site No.7)			Sensible heat, latent heat, and CO2 fluxes	No. 7 station in matrix	2012-05-29 to 2012-09-18	CSAT3 & Li7500A	Calibration and sensor intercomparison	Landscape scale	30 min	10.3972/hiwater.086.2013.db
35	HiWATER: The Multi-Scale Observation Experiment on Evapotranspiration over heterogeneous land surfaces (MUSOEXE) Dataset—flux observation matrix (an eddy covariance system of site No.8)			Sensible heat, latent heat, and CO2 fluxes	No. 8 station in matrix	2012-05-28 to 2012-09-21	CSAT3 & Li7500	Calibration and sensor intercomparison	Landscape scale	30 min	10.3972/hiwater.087.2013.db
36	HiWATER: The Multi-Scale Observation Experiment on Evapotranspiration over heterogeneous land surfaces (MUSOEXE) Dataset—flux observation matrix (an eddy covariance system of site No.9)			Sensible heat, latent heat, and CO2 fluxes	No. 9 station in matrix	2012-06-04 to 2012-09-17	Gill & Li7500A	Calibration and sensor intercomparison	Landscape scale	30 min	10.3972/hiwater.088.2013.db
37	HiWATER: The Multi-Scale Observation Experiment on Evapotranspiration over heterogeneous land surfaces (MUSOEXE) Dataset—flux observation matrix (an eddy covariance system of site No.10)			Sensible heat, latent heat, and CO2 fluxes	No. 10 station in matrix	2012-06-04 to 2012-09-17	CSAT3 & Li7500	Calibration and sensor intercomparison	Landscape scale	30 min	10.3972/hiwater.089.2013.db
38	HiWATER: The Multi-Scale Observation Experiment on Evapotranspiration over heterogeneous land surfaces (MUSOEXE) Dataset—flux observation matrix (an eddy covariance system of site No.11)			Sensible heat, latent heat, and CO2 fluxes	No. 11 station in matrix	2012-05-29 to 2012-09-18	CSAT3 & Li7500	Calibration and sensor intercomparison	Landscape scale	30 min	10.3972/hiwater.090.2013.db
39	HiWATER: The Multi-Scale Observation Experiment on Evapotranspiration over heterogeneous land surfaces (MUSOEXE) Dataset—flux observation matrix (an eddy covariance system of site No.12)			Sensible heat, latent heat, and CO2 fluxes	No. 12 station in matrix	2012-05-28 to 2012-09-21	CSAT3 & Li7500	Calibration and sensor intercomparison	Landscape scale	30 min	10.3972/hiwater.091.2013.db
40	HiWATER: The Multi-Scale Observation Experiment on Evapotranspiration over heterogeneous land surfaces (MUSOEXE) Dataset—flux observation matrix (an eddy covariance system of site No.13)			Sensible heat, latent heat, and CO2 fluxes	No. 13 station in matrix	2012-05-27 to 2012-09-20	CSAT3 & Li7500A	Calibration and sensor intercomparison	Landscape scale	30 min	10.3972/hiwater.092.2013.db
41	HiWATER: The Multi-Scale Observation Experiment on Evapotranspiration over heterogeneous land surfaces (MUSOEXE) Dataset—flux observation matrix (an eddy covariance system of site No.14)			Sensible heat, latent heat, and CO2 fluxes	No. 14 station in matrix	2012-05-30 to 2012-09-21	CSAT3 & Li7500	Calibration and sensor intercomparison	Landscape scale	30 min	10.3972/hiwater.093.2013.db
42	HiWATER: The Multi-Scale Observation Experiment on Evapotranspiration over heterogeneous land surfaces (MUSOEXE) Dataset—flux observation matrix (an eddy covariance system of site No.16)			Sensible heat, latent heat, and CO2 fluxes	No. 16 station in matrix	2012-06-06 to 2012-09-17	Gill & Li7500	Calibration and sensor intercomparison	Landscape scale	30 min	10.3972/hiwater.094.2013.db
43	HiWATER: The Multi-Scale Observation Experiment on Evapotranspiration over heterogeneous land surfaces (MUSOEXE) Dataset—flux observation matrix (an eddy covariance system of site No.17)			Sensible heat, latent heat, and CO2 fluxes	No. 17 station in matrix	2012-05-31 to 2012-09-17	CSAT3 & EC150	Calibration and sensor intercomparison	Landscape scale	30 min	10.3972/hiwater.095.2013.db
44	HiWATER: The Multi-Scale Observation Experiment on Evapotranspiration over heterogeneous land surfaces (MUSOEXE) Dataset—flux observation matrix (large aperture scintillometer at site No.1)		LAS	Sensible heat flux	No. 1 site (north: 100.352°E, 38.884°N; south: 100.351°E, 38.855°N)	2012-06-07 to 2012-09-19 for BLS900 and 2012-06-16 to 2012-09-19 for zzlas	BLS900 & zzlas	Calibration and intercomparison	km scale	30 min	10.3972/hiwater.102.2013.db
45	HiWATER: The Multi-Scale Observation Experiment on Evapotranspiration over heterogeneous land surfaces (MUSOEXE) Dataset—flux observation matrix (large aperture scintillometer at site No.2)			Sensible heat flux	No. 2 site (north: 100.363°E, 38.883°N; south: 100.362°E, 38.857°N)	2012-06-07 to 2012-09-19 for BLS900 and 2012-06-18 to 2012-09-19 for BLS450	BLS900 & BLS450	Calibration and intercomparison	km scale	30 min	10.3972/hiwater.103.2013.db
46	HiWATER: The Multi-Scale Observation Experiment on Evapotranspiration over heterogeneous land surfaces (MUSOEXE) Dataset—flux observation matrix (large aperture scintillometer at site No.3)			Sensible heat flux	No. 3 site (north: 100.373°E, 38.883°N; south: 100.372°E, 38.856°N)	2012-06-06 to 2012-09-20 for BLS900 and 2012-06-19 to 2012-09-20 for Kipp&zonen	BLS900 and Netherland Kipp&zonen	Calibration and intercomparison	km scale	30 min	10.3972/hiwater.104.2013.db
47	HiWATER: The Multi-Scale Observation Experiment on Evapotranspiration over heterogeneous land surfaces (MUSOEXE) Dataset—flux observation matrix (large aperture scintillometer at site No.4)			Sensible heat flux	No. 4 site (north: 100.379°E, 38.861°N; south: 100.369°E, 38.847°N)	2012-06-02 to 2012-09-22 for BLS450 and 2012-06-11 to 2012-09-20 for zzlas	BLS450 & zzlas	Calibration and intercomparison	km scale	30 min	10.3972/hiwater.105.2013.db
48	HiWATER: The Multi-Scale Observation Experiment on Evapotranspiration over heterogeneous land surfaces (MUSOEXE) Dataset—Flux Observation Matrix (stable isotopic observations)		Stable isotopic observation	Atmospheric water vapor D/H and 18O/16O isotopic and flux ratio, D/H and 18O/16O isotopic ratios of water in soil and in corn xylem	Daman superstation (continuous) and No. 13 station (during airborne mission)	2012-05-27 to 2012-09-21	H218O, HDO and H2O analyzer (Model L1102-i, Picarro Inc.)	Picarro analyzer were calibrated during every 3 h switching cycle using a two-point concentration interpolation procedure	Leaf, individual plant scales	2 min per intake and block-averaged to hourly; sampling frequency of soil and xylem was 1–3 days	10.3972/hiwater.108.2013.db
49	HiWATER: The Multi-Scale Observation Experiment on Evapotranspiration over heterogeneous land surfaces (MUSOEXE) Dataset—flux observation matrix (Thermal Dissipation sap flow velocity Probe)		Sap flow	Sap flow rate, sap flow flux, and daily transpiration	Matrix area	2012-06-14 to 2012-09-21	TDP30 (thermal dissipation sap flow velocity probe)	careful check and selection	Individual plant scale	10 min	10.3972/hiwater.106.2013.db
50	HiWATER: The Multi-Scale Observation Experiment on Evapotranspiration over heterogeneous land surfaces (MUSOEXE) Dataset—Flux Observation Matrix (soil moisture obtained via COSMOS)		Soil moisture obtained via COSMOS	Fast neutron counts, corrected fast neutron counts, volumetric soil moisture	Near Daman superstation	2012-06-01 to 2012-09-20	COSMOS	With calibration and air pressure correction	Footprint diameter is 700 m	1 h	10.3972/hiwater.107.2013.db
51	HiWATER: WATERNET soil moisture and LST observation dataset in the middle reaches of the Heihe River Basin	Sensor network	WATERNET	Soil moisture, soil temperature, LST	Matrix area	2012-05-12 to 2012-09-20	Hydra Probe II, SI-111	Two-point calibration for all sensors	Sub-meter to 1-km scale	10 min & 1 min is activated during 00:00-04:30, 08:00-18:00 and 21:00-24:00 BJT	10.3972/hiwater.118.2013.db
52	HiWATER: SoilNET soil moisture observation dataset in the middle reaches of the Heihe river basin		SoilNET	Soil moisture, soil temperature	A 1´1 km^2 grid in the matrix area	2012-06-22 to 2013-03-16	SPADE	Two-point calibration for all sensors	Meter to 100 m	10 min	10.3972/hiwater.120.2013.db
53	HiWATER: BNUNET soil moisture and LST observation dataset in the middle reaches of the Heihe River Basin		BNUNET	Soil moisture, soil temperature	Matrix area	2012-05-12 to 2012-09-16	BNUNET-TEMP, BNUNET-SM	All sensor calibrated	Meter to 1-km scale	10 min	10.3972/hiwater.119.2013.db
54	HiWATER: Dataset of LAINet leaf area index observations in the middle reaches of the Heihe River Basin		LAINet	LAI	Matrix area	2012-06-25 to 2012-08-24	LAINet	Intercomparison with LAI2000	Sub-meter scale	5 days	10.3972/hiwater.057.2013.db
55	HiWATER: Dataset of investigation on channel flow and socio-economy in the middle reaches of the Heihe River Basin	Irrigation	Irrigation water		Yingke and Daman irrigation districts	2008; 2010; 2011; 2012-05-22; 2012-06-18; 2012-07-16; 2012-08-08;		NA	Water management stations		10.3972/hiwater.125.2013.db
56	HiWATER: Dataset of measurements on channel flow in the middle reaches of the Heihe River Basin		Channel flow		Yingke and Daman irrigation districts	2012-05-22 2012-06-18 2012-07-16	Flow meter named Flowatch	Intercomparison with channel flow	Lateral channel		10.3972/hiwater.123.2013.db
57	HiWATER: Dataset of photosynthesis observed by LI-6400 in the middle reaches of the Heihe River Basin	Photosynthesis process	Photosynthesis	NA	Daman superstation; Pingchuan in Linze	2012-05-17 to 2012-09-15	LI-6400XT	Intercomparison between two instruments	Leaf scale	NA	10.3972/hiwater.046.2013.db
58	HiWATER: Dataset of soil respiration rate observed in the middle reaches of the Heihe River Basin		Soil respiration	Soil respiration rate	Daman superstation	2012-06-19 to 2012-09-15	LI-Cor8100A	NA	Individual plant scale	30 min	10.3972/hiwater.126.2013.db
59	HiWATER: Dataset of the Chamber Soil Respiration in the middle reaches of the Heihe River Basin		Soil respiration	NA	Daman superstation, Bajitan gobi desert, Huazhaizi desert steppe, Shenshawo sandy desert, Zhangye wetland, and No. 17 stations	2012-06-16 to 2012-09-22	Static chamber/gas chromatograph	NA	Meter scale	10 days	10.3972/hiwater.035.2013.db
60	HiWATER: Dataset of the Portable Soil Respiration in the middle reaches of the Heihe River Basin		Soil respiration	NA	Daman superstation, No. 1 & 17 stations	2012-06-06 to 2012-08-20	LI-Cor8100	NA	Individual plant scale	5 days	10.3972/hiwater.034.2013.db
61	HiWATER: Dataset of crop leaf stomatal conductance observed in the middle reaches of the Heihe River Basin		Stomatal conductance	NA	Daman superstation, the Shiqiao sample site, the soil moisture control experimental field in Daman county	2012-05-17 2012-09-15	Leaf porometer	NA	Stomatal & leaf scales	5 days	10.3972/hiwater.127.2013.db
62	HiWATER: Dataset of vegetation FPAR in the middle reaches of the Heihe River Basin	Vegetation dynamics	fpar	PAR	Matrix area	2012-05-24 to 2012-07-19	Accupar	NA	Individual plant scale	1–5 day(s)	10.3972/hiwater.044.2013.db
63	HiWATER: Dataset of vegetation LAI measured by LAI2000 in the middle reaches of the Heihe River Basin		LAI	LAI	Matrix area	2012-05-24 to 2012-09-20	LAI-2000	NA	Individual plant scale	5–10 day(s)	10.3972/hiwater.058.2013.db
64	HiWATER: Dataset of Fractional Vegetation Cover in the middle reaches of the Heihe River Basin		FVC	FVC	FEA	2012-05-28 to 2012-09-14	Digital photography	NA	Individual plant scale	5–10 day(s)	10.3972/hiwater.043.2013.db
65	HiWATER: Dataset of investigation on crop phrenology and field management in the middle reaches of the Heihe River Basin		Crop phenology	Crop type, crop name, seed time, seed type, plant span, row span, field area, germination time, three leaves period, seven leaves period, farming way, farming time, irrigation time, irrigation water volume, fertilization time, fertilization type, and fertilization rate	Matrix area	June, 2012	Surveying	NA	Individual plant scale	Growing season	10.3972/hiwater.124.2013.db
66	HiWATER: Dataset of crop height observed in the middle reaches of the Heihe River Basin		Crop height	Crop height	Matrix area	2012-05-17 to 2012-09-15	Steel tape	NA	Individual plant scale	5–10 days	10.3972/hiwater.121.2013.db
67	HiWATER: Dataset of crop leaf chlorophyll content observed in the middle reaches of the Heihe River Basin		Chlorophyll	Crop leaf chlorophyll content	Matrix area	2012-05-17 to 2012-09-15	SPAD	NA	Individual plant scale	5–10 days	10.3972/hiwater.128.2013.db
68	HiWATER: Dataset of sun photometer observations in the middle and upper reaches of the Heihe River Basin in 2012	Atmospheric sounding	Sun photometer	Aerosol optical depth and water vapor contents in different VNIR bands	Daman superstation	2012-06-01 to 2012-09-20	CE318-NE	Intercomparison between two instruments	NA	1 min	10.3972/hiwater.022.2013.db
69	HiWATER: Dataset of GPS radiosonde sounding observations in the middle and upper reaches of the Heihe River Basin in 2012		GPS radiosonde	Pressure, temperature, relative humidity, wind speed, and wind direction profiles	KEA	2012-06-01 to 2012-08-31	RS92-SGP (Vaisala inc.), CF-06-A (Changfeng Micro-Electroinics)	NA		Concurrent with airborne missions	10.3972/hiwater.023.2013.db
70	HiWATER: Dataset of Scintec Flat Array Sodar in the villiage of Wuxing, Xiaoman Town		Acoustic wind profile	Wind direction and wind speed profile	Daman superstation	2012-06-21 to 2012-09-15	MFAS Flat Array Sodar	Intercomparison with ASW	10 m	30 min	10.3972/hiwater.025.2013.db
71	HiWATER: Dataset of soil parameters in the middle reaches of the Heihe River Basin	Soil parameters	Soil texture and hydraulic parameters	Soil texture, porosity, bulk density, saturated hydraulic conductivity, soil organic matter	Soil samples were collected at all AMS stations	2012-05-20 to 2012-07-10	Soil texture: Microtrac laser particle analyzer; Porosity: Ring sampler law; Bulk density: Ring sampler law; Saturated hydraulic conductivity: Hydrostatic head method; Soil organic matter: Total organic carbon analyzer (TOC-VCPH)	NA	Sub-meter scale	No temporal variation	10.3972/hiwater.147.2013.db
72	HiWATER: Dataset of emissivity in the middle reaches of the Heihe River Basin in 2012	Ground-based remote sensing	Emissivity	Emissivity spectrum range from 8 to 14 μm (with spectral resolution of 4 cm^-1) for typical land types	KEA	2012-05-25 to 2012-07-18	102F portable Fourier transform infrared spectrometer and handheld infrared thermometer	NA	Meter scale	--	10.3972/hiwater.042.2013.db
73	HiWATER: Dataset of thermal infrared spectrum observed by BOMEM MR304 in the middle reaches of the Heihe River Basin		Emissivity	Emissivity spectrum (8-14 μm) for typical land types	KEA	2012-5-29 to 2012-07-13	BOMEM MR304 FTIR, Mikron M340 blackbody, BODACH BDB blackbody, diffused golden plate, Fluke 50-series II thermometer	NA	Meter scale	--	10.3972/hiwater.041.2013.db
74	HiWATER: Dataset of the spectral reflectance in the middle reaches of the Heihe River Basin		Spectral reflectance	Spectral reflectance at VNIR bands for typical land types	FEA	2012-6-15 to 2012-07-11	SVC-HR1024, ASD Field Spec 3	Intercomparison between two instruments	Meter scale	--	10.3972/hiwater.037.2013.db
75	HiWATER: Dataset of soil moisture measurements synchronizing with TerraSAR-X satellite overpassing in the Daman Superstation		Soil mosture	Soil mosture	KEA	2012-6-04 to 2012-06-26	Steven Hydro probes	Intercomparison	Meter scale		10.3972/hiwater.047.2013.db
76	HiWATER: Dataset of soil moisture measurements synchronizing with airborne PLMR mission		Soil mosture	Soil mosture	FEA	2012-06-28 to 2012-08-02	Steven Hydro probes	Intercomparison	Meter scale		10.3972/hiwater.052.2013.db
77	HiWATER: Dataset of airborne microwave radiometers (L bands) mission in the middle reaches of the Heihe River Basin on 30 June, 2012	Airborne remote sensing	Microwave radiometer	TB at H & V polarizations	FEA	2012-6-30	PLMR	‘warm’ and ‘cold’ calibration	750 m	N/A	10.3972/hiwater.013.2013.db
78	HiWATER: Dataset of airborne microwave radiometers (L bands) mission in the river way of middle reaches of the Heihe River Basin on 3 July, 2012			TB at H & V polarizations	Along riverway in KEA	2012-7-3	PLMR	‘warm’ and ‘cold’ calibration	100 m	N/A	10.3972/hiwater.014.2013.db
79	HiWATER: Dataset of airborne microwave radiometers (L bands) mission in the river way of middle reaches of the Heihe River Basin on 4 July, 2012			TB at H & V polarizations	Along riverway in KEA	2012-7-4	PLMR	‘warm’ and ‘cold’ calibration	300 m	N/A	10.3972/hiwater.015.2013.db
80	HiWATER: Dataset of airborne microwave radiometers (L bands) mission in the middle reaches of the Heihe River Basin on 5 July, 2012			TB at H & V polarizations	Along riverway in KEA & Daman irrigation district	2012-7-5	PLMR	‘warm’ and ‘cold’ calibration	600 m	N/A	10.3972/hiwater.016.2013.db
81	HiWATER: Dataset of airborne microwave radiometers (L bands) mission in the middle reaches of the Heihe River Basin on 7 July, 2012			TB at H & V polarizations	FEA	2012-7-7	PLMR	‘warm’ and ‘cold’ calibration	600 m	N/A	10.3972/hiwater.017.2013.db
82	HiWATER: Dataset of airborne microwave radiometers (L bands) mission in the middle reaches of the Heihe River Basin on 10 July, 2012.			TB at H & V polarizations	FEA	2012-7-10	PLMR	‘warm’ and ‘cold’ calibration	750 m	N/A	10.3972/hiwater.018.2013.db
83	HiWATER: Dataset of airborne microwave radiometers (L bands) mission in the middle reaches of the Heihe River Basin on 26 July, 2012			TB at H & V polarizations	FEA	2012-7-26	PLMR	‘warm’ and ‘cold’ calibration	700 m	N/A	10.3972/hiwater.019.2013.db
84	HiWATER: Dataset of airborne microwave radiometers (L bands) mission in the middle reaches of the Heihe River Basin on 2 August, 2012			TB at H & V polarizations	FEA	2012-8-2	PLMR	‘warm’ and ‘cold’ calibration	700 m	N/A	10.3972/hiwater.021.2013.db
85	HiWATER: Wide-angle Infrared Dual-mode line/area Array Scanner, WIDAS (26 July, 2012)		Wide-angle infrared dual-mode line/area array scanner	Multi-angular VNIR reflectances (5 bands) and TB at thermal band	FEA	2012-7-26	WIDAS	In-lab calibration, atmospheric and geometric corrections	1 m for VNIR bands; 4.8 m for thermal band		10.3972/hiwater.002.2013.db
86	HiWATER: Wide-angle Infrared Dual-mode line/area Array Scanner, WIDAS (1 August, 2012)			Multi-angular VNIR reflectances (5 bands) and TB at thermal band	FEA	2012-8-1	WIDAS	In-lab calibration, atmospheric and geometric corrections	0.4 m for VNIR bands; 2 m for thermal band		10.3972/hiwater.003.2013.db
87	HiWATER: Wide-angle Infrared Dual-mode line/area Array Scanner, WIDAS (2 August, 2012)			Multi-angular VNIR reflectances (5 bands) and TB at thermal band	FEA	2012-8-2	WIDAS	In-lab calibration, atmospheric and geometric corrections	1.3 m for VNIR bands; 6.3 m for thermal band		10.3972/hiwater.004.2013.db
88	HiWATER: Wide-angle Infrared Dual-mode line/area Array Scanner, WIDAS (3 August, 2012)			Multi-angular VNIR reflectances (5 bands) and TB at thermal band	FEA	2012-8-3	WIDAS	In-lab calibration, atmospheric and geometric corrections	0.4 m for VNIR bands; 2 m for thermal band		10.3972/hiwater.001.2013.db
89	HiWATER: visible and near-infrared hyperspectral radiometer (29 June, 2012)		VNIR & SWIR hyperspectral radiometer	Reflectance spectrum	FEA	2012-6-29	CASI/SASI	In-lab calibration, atmospheric and geometric corrections	1 m for VNIR bands; 2.4 m for SWIR bands		10.3972/hiwater.012.2013.db
90	HiWATER: visible and near-infrared hyperspectral radiometer (7 July, 2012)			Reflectance spectrum	FEA	2012-7-7	CASI/SASI	In-lab calibration, atmospheric and geometric corrections	1 m for VNIR bands; 2.4 m for SWIR bands		10.3972/hiwater.011.2013.db
91	HiWATER: Thermal-Infrared Hyperspectral Radiometer (30 June, 2012)		Thermal-Infrared hyperspectral radiometer	LST and emissivity	FEA	2012-6-30	TASI	In-lab calibration, atmospheric and geometric corrections	3 m		10.3972/hiwater.005.2013.db
92	HiWATER: Thermal-Infrared Hyperspectral Radiometer (4 July, 2012)			LST and emissivity	Along riverway in KEA, FEA	2012-7-4	TASI	In-lab calibration, atmospheric and geometric corrections	3 m		10.3972/hiwater.006.2013.db
93	HiWATER: Thermal-Infrared Hyperspectral Radiometer (10 July, 2012)			LST and emissivity	FEA	2012-7-10	TASI	In-lab calibration, atmospheric and geometric corrections	3 m		10.3972/hiwater.007.2013.db
94	HiWATER: Airborne LiDAR-DEM data production in the middle reaches of the Heihe River Basin	Airborne remote sensing data products	DEM		KEA	2012-7-19	Leica ALS70	Parameter calibration, automatic classification of point cloud density and manual editing	4 points m^-2		10.3972/hiwater.010.2013.db
95	HiWATER: Airborne LiDAR-DSM data production in the middle reaches of the Heihe River Basin		DSM		KEA	2012-7-19	Leica ALS70	Parameter calibration, automatic classification of point cloud density and manual editing	4 points m^-2		10.3972/hiwater.149.2013.db
96	HiWATER: the albedo in the middle reaches of the Heihe River Basin (29 June, 2012)		Albedo		Matrix area	2012-6-29	CASI	NA	5 m		10.3972/hiwater.167.2014.db
97	HiWATER: Land cover map in the Core Experimental Area of flux observation matrix		Vegetation type		Matrix area	June, 2012	CASI	The accurcay=84.6% and Kappa coefficient=0.83.	1 m; 2 m		10.3972/hiwater.156.2014.db
98	HiWATER: Vegetation height product in the middle reaches of the Heihe River Basin (19 July, 2012)		Vegetation height		KEA	2012-7-19	Leica ALS70	NA	1 m		10.3972/hiwater.172.2014.db
99	HiWATER: Land surface temperature product in the middle reaches of the Heihe River Basin (30 June, 2012)		LST		FEA	2012-6-30	TASI	RMSE<1.5 K	3 m		10.3972/hiwater.168.2014.db
100	HiWATER: Land surface temperature product in the middle reaches of the Heihe River Basin (10 July, 2012)				Along riverway in KEA, FEA	2012-7-10	TASI	RMSE<1.5 K	3 m		10.3972/hiwater.170.2014.db
101	HiWATER: Land surface temperature product in the middle reaches of the Heihe River Basin (4 July, 2012)				FEA	2012-7-4	TASI	RMSE<1.5 K	3 m		10.3972/hiwater.169.2014.db
102	HiWATER: Dataset of retrieved soil moisture products using PLMR brightness temperatures in the middle reaches of the Heihe River Basin		Soil moisture		KEA	2012-06-30 to 2012-08-02	PLMR	RMSE<0.05 m^3 m^-3	700 m		10.3972/hiwater.174.2014.db
		Note 1	Air temperature and humidity profile, wind speed and wind direction profile(3, 5, 10, 15, 20, 30, 40 m), air pressure, precipitation, four-component radiation (incoming shortwave radiation, outgoing shortwave radiation, incoming longwave radiation, outgoing longwave radiation), infrared LST, photosynthetically active radiation, average soil temperature, soil heat flux, soil temperature profile (0,−0.02, −0.04, −0.1, −0.2, −0.4, −0.8, −1.2, −1.6 m), soil moisture profile(−0.02, −0.04, −0.1, −0.2, −0.4, −0.8, −1.2, −1.6 m) (Xu *et al.*^13^; Liu *et al.*, 2015)								
		Note 2	Air temperature and humidity (AV-14TH, Avalon), wind speed and wind direction (windsonic,Gill),air pressure (CS100, Campbell), precipitation (TE525MM,Campbell), four-component radiation (PSP&PIR, Eppley), infrared LST (SI-111, Apogee), photosynthetically active radiation (LI-190SB, Li-cor), average soil temperature (TCAV, Campbell), soil heat flux (HFP01SC, Hukseflux), soil temperature profile (AV-10T, Avalon), and soil moisture profile (CS616, Campbell) (Liu *et al.*, 2015)								
		Note 3	Air temperature and humidity profile (5 & 10 m), wind speed profile (5 & 10 m), wind direction, air pressure, precipitation, four-component radiation, LST, soil heat flux, soil temperature profile (0, −0.02, −0.04, −0.1, −0.2, −0.4, −0.6, and −1.0 m), and soil moisture profile (−0.02, −0.04, −0.1, −0.2, −0.4, −0.6, and −1.0 m) (Xu *et al.*^13^; Liu *et al.*, 2015). At sites 3,9,10,16, no wind direction and air pressure, soil temperature (0,−0.02,−0.04 m), soil moisture(−0.02, −0.04 m); only net radiation in site 16; at sites 1,4,5,7,8,11-14,16,17, air temperature and humidity (5 m), wind speed (10 m); at the Huazhaizi station, wind speed profile (0.48, 0.98, 1.99, 2.99 m), air temperature and humidity profile (1.00, 1.99, 2.99 m), soil temperature profile (0, −0.02, −0.04, −0.1, −0.18, −0.26, −0.34, −0.42, −0.50 m), soil moisture profile (−0.02, −0.04, −0.1, −0.18, −0.26, −0.34 m); at the wetland station, no soil moisture sensor was installed.								
		Note 4	The sampling frequencies of many observations were much higher. The temporal resolutions indicated in this table are those of the analyzed datasets.								

**Table 2:** Characteristics of airborne sensors used in the HiWATER 2012 IOP airborne campaigns.

**Airborne sensor**	**Major sensor characteristics**	**Minimum required performance limit**	**Full width at half maximum**	**Angle(s) of observation**
LiDAR (Leica ALS70)+CCD camera	Maximum flight altitude: 5,000 m; FOV: 75°; waveform: first, second and third return intensities; vertical placement accuracy: 5–30 cm; Integral digital camera: 1,280×1,024 pixels; CCD with four bands: 420–520 nm, 500–580 nm, 565–660 nm, and 780–880 nm	Vertical accuracy of 2 cm; full waveform; sampling interval of 1 ns	LiDAR: Pulse temporal width of 9 ns at <200 kHz and 4 ns at >200 kHz; CCD camera with four bands: 100, 80, 95 and 100 nm	Nadir
CASI-1500 VNIR imaging spectrometer	Spectral range: 380–1050 nm; 1,500 across-track pixels; 288 continuous spectral bands; spectral band width: 2.3 nm; frame frequency: 14; FOV: 40°	Spectral resolution of ≤5 nm; spatial resolution of 1–5 m	2.3 nm	Nadir
SASI-600 hyperspectral SWIR imaging system	Spectral range: 950–2,450 nm; 600 across-track pixels; 101 spectralbands; bandwidth: 15 nm; FOV: 40°	Spectral resolution of ≤10 nm; spatial resolution of 1–30 m	15 nm	Nadir
TASI-600 pushbroom hyperspectral thermal sensor system	Spectral range: 8,000–11,500 nm; 600 spatial pixels; 32 bands; bandwidth: 110 nm; FOV:40°	NETD of 0.2 K; spatial resolution of 5–10 m	55 nm	Nadir
WiDAS (Wide-angle infrared Dual-mode line/area Array Scanner)	Thermal imaging camera: 7.5–14 μm; 640×480 pixels; a wide-angle lens (68°×54°); Two CCD cameras with four visible bands of 400–500 nm, 500–590 nm, 590–670 nm, and 670–850 nm; one near infrared band of 850–1000 nm; 1,360×1,024 pixels; FOV: 50°	NETD of 0.2 K; spatial resolution of 5–10 m	CCD camera with five bands: 110, 80, 90, 10 and 140 nm; thermal camera: 7.5–14 μm	Thermal imaging camera: forward inclination angle of 12°; two CCD cameras: forward inclination angles of 9° and 36°
PLMR (Polarimetric L-band Multibeam Radiometer)	Frequency: 1.413 GHz; bandwidth: 24 MHz; resolution: 1 km (flight altitude: 3 km AGL); adjustable incident angle: ±7.5°, ±21.5°, or ±38.5°; sensitivity<1 K; polarization: V/H	Spatial resolution of 100 m–1 km	24 MHz	Nadir, ±7.5°, ±21.5°, and ±38.5°
VNIR, visible and near infrared; SWIR, shortwave infrared; NETD, noise equivalent temperature difference.				

**Table 3:** Summary of released HiWATER 2012 IOP datasets.

**Data**	**Dataset quantity**	**Data size**	**Data format**
Flux observation matrix	50	158.6 MB	EXCEL
Ecohydrological sensor network	4	1749.0 MB	EXCEL
Other ground-based observations	22	16.2 GB	EXCEL
Airborne missions and airborne remote sensing products	26	781.8 GB	GeoTIFF, ENVI hdr & img
Total	102	799.9 GB	

**Table 4:** High-resolution satellite remote sensing data obtained during HiWATER 2012 IOP.

**Remote sensors**	**Sensor type**	**Spatial resolution**	**Acquisition times**
ASTER	VNIR/TIR	15–90 m	2012-05-30, 2012-06-15, 2012-06-24, 2012-07-10, 2012-08-02, 2012-08-11, 2012-08-18, 2012-08-27, 2012-09-03, 2012-09-12, 2012-09-19, 2012-09-28
COSMO-SkyMed	SAR	15 m	2012-07-25, 2012-07-28, 2012-08-02
Landsat ETM+	VNIR/TIR	15–60 m	2012-04-05, 2012-04-21, 2012-05-07, 2012-06-24, 2012-07-10
PROBA CHRIS	Hyperspectral imager	30 m	2012-06-21, 2012-06-29, 2012-07-10, 2012-08-27
Radarsat-2	SAR	8 m	2012-07-06
TerraSAR-X	SAR	3 m	2012-05-24, 2012-06-04, 2012-06-26, 2012-07-07, 2012-07-29, 2012-08-09, 2012-08-14, 2012-08-25
ZY-3	VNIR	1 m	2012-08-25, 2012-09-03, 2012-09-08, 2012-09-13, 2012-09-18, 2012-09-23, 2012-09-28, 2012-10-03, 2012-10-13, 2012-10-18, 2012-10-22, 2012-11-01, 2012-11-11, 2012-11-21
